# *Lactobacillus plantarum* TW1-1 Alleviates Diethylhexylphthalate-Induced Testicular Damage in Mice by Modulating Gut Microbiota and Decreasing Inflammation

**DOI:** 10.3389/fcimb.2019.00221

**Published:** 2019-06-26

**Authors:** Xiaozhu Tian, Zhengsheng Yu, Pengya Feng, Ze Ye, Rong Li, Juyuan Liu, Junping Hu, Apurva Kakade, Pu Liu, Xiangkai Li

**Affiliations:** ^1^Ministry of Education Key Laboratory of Cell Activities and Stress Adaptations, School of Life Science, Lanzhou University, Lanzhou, China; ^2^Key Laboratory for Reproductive Medicine and Embryo, The Reproductive Medicine Special Hospital of the First Hospital of Lanzhou University, Lanzhou, China

**Keywords:** diethylhexylphthalate toxicity, *Lactobacillus plantarum* TW1-1, oxidative stress, inflammatory response, intestinal permeability, gut microbiota

## Abstract

Diethylhexylphthalate (DEHP), acting as an endocrine disruptor, disturbed reproductive health. Here, we evaluated the effects of *Lactobacillus plantarum* TW1-1 (*L. plantarum* TW1-1) on DEHP-induced testicular damage in adult male mice. Results showed that oral supplementation of *L. plantarum* TW1-1 significantly increased the serum testosterone concentration, enhanced the semen quality, and attenuated gonad development defects in DEHP-exposed mice. *L. plantarum* TW1-1 also alleviated DEHP-induced oxidative stress and inflammatory responses by decreasing the mRNA expression and serum protein concentration of different inflammatory factors [tumor necrosis factor-α, interleukin (IL)-1β and IL-6]. Furthermore, *L. plantarum* TW1-1 significantly reduced DEHP-induced intestinal hyper-permeability and the increase in the serum lipopolysaccharide level. Gut microbiota diversity analysis revealed that *L. plantarum* TW1-1 shifted the DEHP-disrupted gut microbiota to that of the control mice. At phylum level, *L. plantarum* TW1-1 reversed DEHP-induced *Bacteroidetes* increase and *Firmicutes* decrease, and restored *Deferribacteres* in DEHP-exposed mice. Spearman's correlation analysis showed that *Bacteroidetes, Deferribacteres*, and *Firmicutes* were associated with DEHP-induced testicular damage. In addition, the ratio of *Firmicutes* to *Bacteroidetes* (Firm/Bac ratio) significantly decreased from 0.28 (control group) to 0.13 (DEHP-exposed group), which was restored by *L. plantarum* TW1-1 treatment. Correlation analysis showed that the Firm/Bac ratio was negatively correlated with testicular damage and inflammation. These findings suggest that *L. plantarum* TW1-1 prevents DEHP-induced testicular damage via modulating gut microbiota and decreasing inflammation.

## Introduction

Diethylhexylphthalate (DEHP) is the most commonly used phthalate for the production of flexible polyvinylchloride. It is present in consumer products, such as food packaging bags, medical devices, or food additives, which are widely used in daily life. However, Phthalates can easily leach out and enter environmental cycles. Studies indicate that phthalate acid esters in soil could enter plants and reach the human body or other organisms via food chains, and are potentially carcinogenic and mutagenic (Wang et al., 2015a). According to a study, 0.20–7.11 mg/Kg concentration of DEHP was detected in the soil samples of 23 cities in China. Most of the food and drinking water were also detected to be contaminated by DEHP (Zhang et al., 2015a). Therefore, DEHP has become a significant source of environmental pollution.

Toxicological studies have confirmed that DEHP can act as an endocrine disruptor and reproductive toxicant. DEHP adversely affects the development of male reproductive tract, and reduces semen quality (Kay et al., 2014). Exposure to DEHP has been shown to induce testicular damage by reducing the thickness of seminiferous epithelium and the number of cell layers (Zhang et al., 2014). DEHP has been proved to be a concern for human reproductive health, which leads to the decrease of sperm count and motility, and sperm DNA damage in a human body (Klinefelter et al., 2012). In addition, DEHP-induced testicular damage is associated with the increase of reactive oxygen species (ROS) and lipoperoxidation, and the decrease of antioxidants (Zhang et al., 2014; Abdel-Kawi et al., 2016). A previous study has also provided an evidence that DEHP inhibits testosterone hormone production and causes oxidative stress (Ha et al., 2016). Therefore, oxidative stress has been recognized as a critical mechanism underlying DEHP-induced testicular injury. Although many natural antioxidants, such as lycopene, quercetin, and curcumin (Zhang et al., 2014; Abdel-Kawi et al., 2016; Bahrami et al., 2018b), have been used to prevent DEHP-induced testicular toxicity though inhibiting oxidative stress. The exact mechanisms of DEHP-induced reproductive toxicity are still unclear.

The emerging researches revealed the relationship between microbial communities and environmental factors, including diet and environmental pollutants (Jin et al., 2017). Diethyl phthalate (DEP), a DEHP-like compound, at a very low dose (0.1735 mg/kg/day), was reported to alter the overall gut bacterial composition in rats (Hu et al., 2016). According to literatures, environmental pollutants could change the composition of gut microbiota, and result in a series of disorders, including energy metabolism, immune system, neurodevelopment, and reproductive development defects or other toxic symptoms (Jin et al., 2015; Zhang et al., 2015b). It is well known that the gut microbiota regulates many physiological functions and plays a very important role in maintaining host health. Previous study showed testosterone level decreased in germ free mice (Nomura et al., 1973). Furthermore, a recent research showed that the gut microbiota participated in modulating BTB permeability and regulating testicular endocrine functions (Al-Asmakh et al., 2014). Therefore, the gut microbiota may be associated with gonad development and reproductive health. Until recently, there is no report illustrating the effect of DEHP exposure on gut microbiota. Hence, this study aims to determine whether DEHP influences the composition of gut microbiome in mice and evaluate the association of gut microbiota with DEHP-induced disruption.

Probiotics maintain gut microbiota balance, regulate immune response, and reduce colonization of pathogenic organisms through competitive inhibition of epithelial and mucosal adhesion (Shanahan, 2010). *Lactobacillus* is a well-documented probiotic that has been used for the regulation of immune system and treatment of gastrointestinal diseases. The probiotic strain, *Lactobacillus plantarum* CCFM8610 protects the mice against cadmium toxicity, whereas, *L. plantarum* CCFM8661 alleviates lead-induced toxicity in mice (Zhai et al., 2014). It has been reported that supplementation of *Lactobacillus reuteri* increases the level of testosterone in aging mice (Poutahidis et al., 2014) and *Lactobacillus rhamnosus* enhances fish backbone calcification and reproduction (Avella et al., 2012; Carnevali et al., 2013). Hence, gut microbial remediation by probiotics is becoming a new perspective for maintaining the health immune system, physiology, reproduction, and nutrient metabolism. Here, we want to investigate whether probiotic could be used as a remediation strategy to diminish environmental pollutant exposure induced physiological impairment in mice.

*L. plantarum* TW1-1 (*L. plantarum* TW1-1), isolated from a fermented milk product, has been recently reported to have anti-inflammatory and anti-oxidative stress activities (Wu et al., 2017). Here, we evaluated the protective effects of *L. plantarum* TW1-1 on DEHP-induced testicular damage in mice through examining inflammatory markers, oxidative stress response, intestinal function and gut microbiota, and disclosed the relationship between reproductive health and gut microbiota. Our results would provide a novel therapeutic approach for environmental pollutants-induced reproductive toxicity.

## Materials and Methods

### Preparation of *L. plantarum* TW1-1 Suspension

*L. plantarum* TW1-1 (KJ026561), isolated from a fermented milk product, was generously provided by Dr. Jian Kong (Shandong University, Jinan, China) and cultured in Man, Rogosa, and Sharpe broth (Beijing Solarbio Science and Technology, Beijing, China) at 37°C in aerobic conditions. After incubation for 24 h, cultures were centrifuged at 8,000 rpm for 5 min, washed three times with normal saline (NS), and then *L. plantarum* TW1-1 was resuspended in NS. *L. plantarum* TW1-1 suspension was harvested after filtering through 0.22 μm filters when its density reached 5 ×10^9^ colony-forming units/ mL, and later was stored at 0–4°C for further use in a week.

### Experimental Animals and Protocols

**Figure 1:**
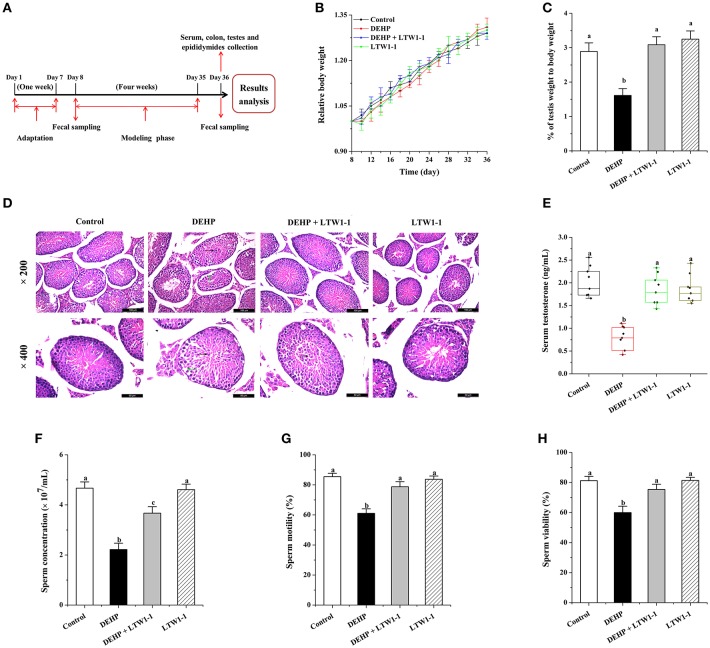
Effects of *L. plantarum* TW1-1 treatment on mouse growth and development in the different groups. **(A)** Experimental design. **(B)** The relative body weight curve of the four groups of mice during the 4 weeks of intervention. The relative body weight was calculated as the percentage of baseline weight for each mouse. **(C)** Average testis weight relative to the body weight for each mouse. **(D)** Photomicrographs of Hematoxylin and eosin stained testicular sections. Green arrows indicate the seminiferous tubules with reduced thickness of the germinal epithelium and black arrows indicate an irregular arrangement of germ cells in the seminiferous tubules with exfoliation of germ cells into the lumen. **(E)** Changes in testosterone levels in the different groups. **(F)** Sperm concentration. **(G)** Sperm motility. **(H)** Sperm viability. The data are expressed as mean ± S.E.M. (*n* = 8), different letters represent significant differences between groups by Tukey's test (*P* < 0.05).

The adult male C57BL/6 mice (10-week-old, 25 ± 2 g) were purchased from the Animal Center of Lanzhou University (Lanzhou, China, SCXK (GAN) 2018-0002) and maintained under specific pathogen-free (SPF) conditions in a separate room at 22 ± 1°C and at 40%−60% relative humidity for a week prior to the treatment. All mice ate and drank freely. DEHP was purchased from Sigma-Aldrich Corporation and dissolved in corn oil (100 mg/mL). Animals were randomly divided into four groups (8 mice per group): the control group (Control), the DEHP-exposed group (DEHP), the DEHP and *L. plantarum* TW1-1 administration group (DEHP + LTW1-1), and the *L. plantarum* TW1-1-treated group (LTW1-1). The control group mice received NS (200 μL) following with corn oil (200 μL) administration 1 h later. DEHP group mice were given DEHP (400 mg/kg body weight, 200 μL) 1 h after NS (200 μL) administration. In the DEHP + LTW1-1 group, mice were administered with 200 μL of *L. plantarum* TW1-1 suspension at the final dose of 10^9^ cells 1 h before DEHP (400 mg/kg body weight, 200 μL) administration. LTW1-1 group mice were fed *L. plantarum* TW1-1 at the same dose with DEHP + LTW1-1 group 1 h before corn oil (200 μL) administration (Figure 1A). All solutions were administered intra-gastrically once a day for 4 weeks.

The body weights were measured every other day. Fecal sample from each mouse was collected at baseline and at the end of the experiment, and then stored at −80°C until processing. Also, serum was collected and stored −80°C for further biochemical investigations. All mice were killed by cervical dislocation. The testis and its epididymis were removed immediately after the mice were sacrificed. The epididymis was removed from testis to collect the semen samples, and the testes were immediately removed and weighed. And then, the colon and testes were collected and quickly exposed in liquid nitrogen, and stored at −80°C until processing. The body and testes weight are shown in Table S1. All animal experimental procedures were approved and carried out according to Lanzhou University's Institutional Animal Care and Use Committee guidelines.

### Sperm Characteristics Analysis

The testis and its epididymis were removed immediately after the mice were sacrificed. The semen samples, collected from epididymides, were incubated in pre-warmed phosphate-buffered saline (pH = 7.2) at 37°C for 20 min, and then, sperm parameters including sperm cell concentration, sperm motility, and viability were examined as previously described (Naghdi et al., 2016). Briefly, sperm cell concentration was analyzed under light microscope. Sperm motility was assessed via counting progressive, non-progressive, and immotile sperm after analyzing the recorded films. 5 μL sperm suspension was transferred on a slide and 20 s film was recorded using video camera in five fields from each slide. To assess sperm viability, we performed 5 μL trypan blue staining by mixing 10 μL of the sperm suspension. The proportions of live and dead sperms were recorded under microscope (original magnification: ×400).

### Histopathology

The testes tissues were fixed with 2.5% paraformaldehyde for 24 h, and then, processed to prepare 5 μm paraffin sections for hematoxylin and eosin staining (H&E) (Beijing Solarbio Science and Technology, Beijing, China).

### Biochemical Analysis

The concentrations of glutathione (GSH), lipid peroxidation (LPO) and malondialdehyde (MDA), and activities of super oxide dismutase (SOD) and catalase (CAT) in the serum, testes, and colon were valued using commercial kits (Nanjing Jiancheng Institute of Biotechnology, Nanjing, China) according to the instructions. Serum testosterone, lipopolysaccharide (LPS), tumor necrosis factor (TNF)-α, interleukin (IL)-1β, and IL-6 levels were measured using corresponding enzyme-linked immunosorbent assay (ELISA) kits (Shanghai Enzyme-linked Biotechnology, Shanghai, China) according to the manufacturer's protocols. The DEHP concentrations of the serum and testes were also detected by DEHP ELISA kit (Shanghai Vinhaket Biological Technology Co. Ltd. China) according to the instructions.

### Gene Expression Assay

Total RNA of the testis and colon of each mouse were extracted using RNA extraction kit (TIANGEN Biotech, Shanghai, China). TNF-α, IL-1β, and IL-6 gene expression levels were determined as described previously (Cani et al., 2009). Quantitative real time polymerase chain reaction (PCR) was performed using SYBR green Master Mix (ShineGene Molecular Biotech Inc., Shanghai, China) on instrument (FTC3000, Canada). The sequences of the forward and reverse primers used have been listed in Table S2. The cycling conditions used to amplify the complementary DNA were as follows: denaturation at 95°C for 1 min, 40 cycles at 95°C for 15 s and 1 min at 55°C. All samples were run in triplicate. Mouse gene *GAPDH* was used to normalize the transcript levels of each inflammatory factor. Quantification of mRNA expression was presented as fold changes with setting the value of the control group mice as one.

### Intestinal Permeability Measurement

Intestinal permeability was assessed using 4000 Da fluorescent-dextran-FITC (Dx-FITC; Sigma-Aldrich, USA). Briefly, mice were administered with 200 μL of Dx-FITC (100 mg/mL) after a 5 h fasting period. Four hours after the gavage of mice, 200 μL of blood was collected and centrifuged at 4°C at 1,000 *g* for 10 min. The serum was collected and stored −80°C until use. Intestinal permeability was determined by measuring the amount of Dx-FITC in the serum with a fluorescence spectrophotometer (excitation 485 nm and emission 535 nm) (Cani et al., 2009).

### DNA Extraction and Sequencing of Fecal Bacterial 16S rRNA Genes

The fecal genomic DNA extraction was performed using the DNA extraction kit (TIANGEN Biotech, Shanghai, China). Briefly, 0.2 g fecal samples were used for DNA extraction and the purified DNA were diluted in 50 μL dH_2_O. Quantification of DNA was carried out using Nanodrop 2000 (Thermo Fisher Scientific, USA). The V4 region of the 16S rRNA gene was amplified from the total DNA using the primer pair, 515F-909R (forward: 5′-GTGCCAGCMGCCGCGGTAA-3′; reverse: 5′-CCCCGYCAATTCMTTTRAGT-3′) with unique barcode for each sample and then used for Illumina Miseq sequencing (Nuozhou Biotech Co., Chengdu, China). 10 ng genomic DNA was used for PCR with 30 cycles of 94°C for 40 s, 56°C for 60 s. For each sample, two PCR reactions were conducted and then combined for recovery from the agarose gel. All samples were pooled together with an equal molar amount from each sample. After obtaining the sequencing data, QIIME Pipeline-Version 1.9.0 was performed for raw data screening and the length of sequences <150 bp were removed. The sequences were clustered into operational taxonomic units (OTUs) at a 97% identity threshold. Singleton sequences were filtered out. Next, each sample was rarefied at 11,080 sequences for further analysis. Ribosomal Database Project (RDP) classifier was performed for taxonomy of each OTU from genus to kingdom. Alpha-diversity was generated based on observed species described as Shannon and Simpson indices and rarefaction curves. Detrended correspondence analysis (DCA) was conducted for the comparison of microbial community in each sample using R software (version v3.1.0). Cladograms generated from linear discriminant analysis (LDA) effect size analysis (LEfSe) were used to show the most differentially abundant taxa enriched in gut microbiota. LDA scores showed the differentially abundant taxa represented in cladograms on the basis of LDA score >2 and a significance of α <0.05.

The sequence data reported in this paper have been submitted to NCBI database and the accession number is PRJNA542534.

### Statistical Analysis

The statistical analysis between groups was performed by one-way analysis of variance (ANOVA) followed by the *post-hoc* test (Tukey's test) using SPSS 16.0. Values are expressed as the mean ± S.E.M. *P* < 0.05 indicated statistical significance. Detrended correspondence analysis (DCA) was conducted for the comparison of microbial community in each sample using R software (version v3.1.0). LDA scores showed the differentially abundant taxa represented in cladograms on the basis of LDA score > 2 and a significance of α <0.05.

## Results

### *L. plantarum* TW1-1 Alleviates DEHP-Induced Testicular Damage in Mice

Firstly, the body weight of each mouse was measured, and no significant difference was observed in body weight between the four groups of mice (Figure 1B). However, the testes weight and the percentage of testes weight to body weight in DEHP group decreased dramatically (*P* = 0.0004), whereas mice treated with DEHP and *L. plantarum* TW1-1 together did not show decrease in the testes weight comparing with both the control and the LTW1-1 groups (Table S1 and Figure 1C). This result indicates that DEHP-induced decrease in testis weight was restored by *L. plantarum* TW1-1 supplementation. Histopathological examination showed that DEHP exposure induced testicular damage. The testes sections from the DEHP group mice showed a thickness reduction of the germinal epithelium and irregular arrangement of germ cells with cell exfoliation into the lumen of seminiferous tubules compared with the control mice (Figure 1D). However, *L. plantarum* TW1-1 administration to the DEHP-exposed mice prevented the irregular arrangement of germ cells, although some germ cells were still exfoliated into the lumen, which indicated that *L. plantarum* TW1-1 partially decreased DEHP-induced testicular damage. Furthermore, we found that DEHP exposure significantly decreased serum testosterone concentration (0.76 ± 0.09 vs. 2.00 ± 0.11 ng/mL, *P* < 0.001) (Figure 1E) in comparison with the control group. Testosterone concentration in the serum of the DEHP + LTW1-1 group mice were at the same level with the control (*P* = 0.379). In the LTW-1 group, *L. plantarum* TW1-1 did not influence testosterone concentration (*P* = 0.62), compared with the control mice. Additionally, mice semen quality was also evaluated. DEHP exposure significantly decreased sperm cell concentration, sperm motility and viability, comparing with the control (*P* < 0.001) (Figures 1F–H), which were restrained with the intervention of *L. plantarum* TW1-1. All together, these results suggest that *L. plantarum* TW1-1 alleviates DEHP-induced reproductive toxicity in mice.

### Effects of *L. plantarum* TW1-1 on Oxidative Stress in the Serum, Testes, and Colon, and DEHP Concentration in the Serum and Testes

**Figure 2:**
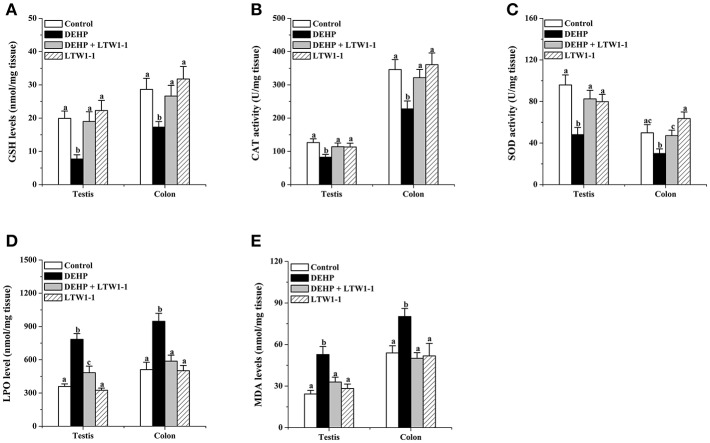
Effects of *L. plantarum* TW1-1 treatment on GSH level, CAT, and SOD activity, and LPO and MDA concentrations in the testes and colon. **(A)** GSH concentration. **(B)** CAT activity. **(C)** SOD activity. **(D)** LPO concentration. **(E)** MDA concentration. The data are expressed as mean ± S.E.M. (n = 8), different letters represent significant differences between groups by Tukey's test (*P* < 0.05).

For the detection of oxidative stress induced by DEPH exposure, we focused on some popular and reliable oxidative stress markers, including GSH, CAT, SOD, LPO, and MDA. As shown in Figures 2A–E, DEHP exposure significantly reduced GSH concentration, CAT, and SOD activities, and increased concentrations of LPO and MDA both in the testes and colon, which means that DEHP caused oxidative stress by reducing the antioxidant status and increasing oxidant status. Moreover, *L. plantarum* TW1-1 supplementation restored the levels of GSH, CAT, SOD, LPO, and MDA in DEHP-exposed mice to those of the control group mice. Comparing with control group, no significant changes were observed in the LTW1-1 group. DEHP-induced oxidative stress and the countering effect of *L. plantarum* TW1-1 were also observed in the serum (Table S3). In addition, DEHP was detected to be present both in the serum and testes. DEHP exposure significantly increased the levels of DEHP in the serum and testes (*P* < 0.001), and *L. plantarum* TW1-1 treatment did not affect this increase (Table 1). All together, our results suggest that *L. plantarum* TW1-1 could alleviates oxidative stress induced by DEHP exposure, even though it do not eliminate the residual DEHP in mice.

**Table 1:** DEHP concentrations in serum and testes in different groups at the end of experiment.

**DEHP (ng/mL)**	**Groups**			
	**Control**	**DEHP**	**DEHP + LTW1-1**	**LTW1-1**
Serum	65.44 ± 5.47^a^	626.34 ± 65.11^b^	578.98 ± 43.62^b^	73.19 ± 7.16^a^
Testes	16.27 ± 3.36^a^	111.29 ± 20.31^b^	138.64 ± 37.78^b^	20.53 ± 6.47^a^

*The data are expressed as mean ± S.E.M. (n = 7–8), different letters represent significant differences between groups by Tukey's test (P <0.001)*.

### *L. plantarum* TW1-1 Treatment Alleviates the Increased Inflammatory Responses in the Serum, Testes, and Colon of DEHP-Exposed Mice

**Figure 3:**
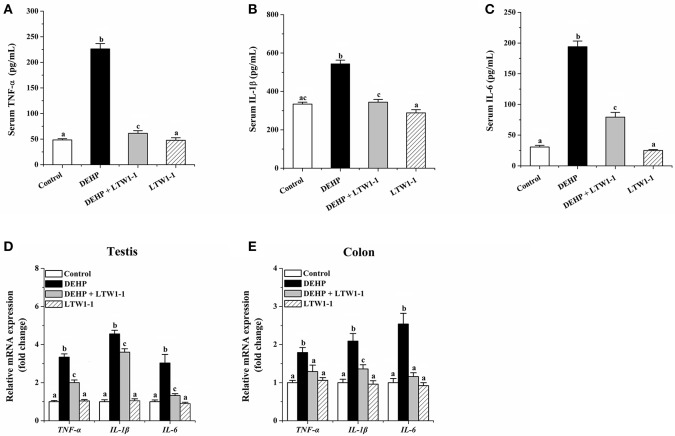
Effects of *L. plantarum* TW1-1 treatment on inflammatory factors in the serum, testes and colon in DEHP-exposed mice. **(A)** Serum TNF-α level. **(B)** Serum IL-1β level. **(C)** Serum IL-6 level. **(D)** Relative mRNA expression of TNF-α, IL-1β, and IL-6 in the colon. **(E)** Relative mRNA expression of TNF-α, IL-1β, and IL-6 in the testis. The levels of TNF-α, IL-1β, and IL-6 were detected by ELISA. Gene expression levels of these cytokines were analyzed by RT-qPCR. The data are expressed as mean ± S.E.M. (*n* = 7–8), different letters represent significant differences between groups by Tukey's test (*P* < 0.05).

On DEHP exposure, as shown in Figures 3A–C, concentrations of TNF-α, IL-1β, and IL-6 in the serum increased obviously, and DEHP-induced high concentrations of those inflammatory molecules were significantly countered by *L. plantarum* TW1-1 administration. In addition, the relative mRNA expression of TNF-α, IL-1β, and IL-6 in the colon and testes were highly increased in DEHP-exposed mice as compared to the control and the LTW1-1 group mice (Figures 3D,E). For those DEHP-exposed mice, additional *L. plantarum* TW1-1 treatment significantly restrained the gene expression of these inflammatory markers, and gene expression levels of TNF-α and IL-6 in the colon were similar to those of the control mice. These results suggest that *L. plantarum* TW1-1 treatment could alleviate systemic and tissue inflammation induced by DEHP exposure.

### *L. plantarum* TW1-1 Pretreatment Prevents the Increase of DEHP-Induced Intestinal Permeability

**Figure 4:**
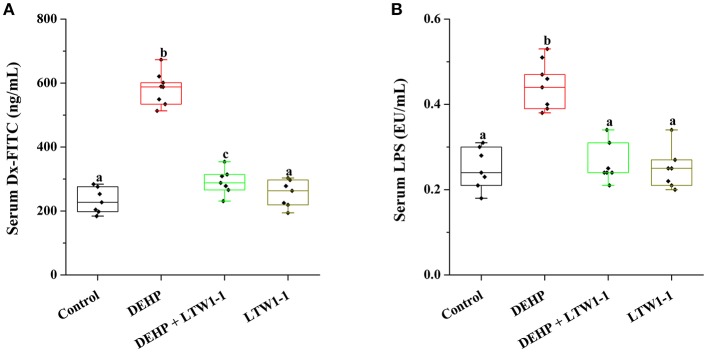
Effects of *L. plantarum* TW1-1 treatment on intestinal permeability in DEHP-exposed mice. **(A)** Serum Dx-FITC levels. **(B)** Serum LPS levels. The LPS levels were detected by ELISA. The data are expressed as mean ± S.E.M. (*n* = 7–8), different letters represent significant differences between groups by Tukey's test (*P* < 0.05).

Dx-FITC and LPS were used to determine the intestinal permeability. For DEHP group mice, compared with the control, the serum Dx-FITC level was significantly increased (Figure 4A, 583.5 ± 18.12 vs. 232.29 ± 14.92 ng/mL, *P* < 0.001), suggesting that DEHP increased intestinal permeability. Additionally, although the serum Dx-FITC concentration in DEHP + LTW1-1 group mice was higher than that of the control mice (*P* = 0.016), *L. plantarum* TW1-1 supplement induced a significant reduction comparing with DEHP group mice (*P* < 0.001). With the application of endotoxin, we determined the serum LPS level of different mice groups. The level of LPS in the DEHP group mice tended to be higher than that in the control mice (0.45 ± 0.02 vs. 0.25 ± 0.02 EU/mL, *P* < 0.001), which further demonstrated the increase in intestinal permeability after DEHP exposure (Figure 4B). Additional *L. plantarum* TW1-1 treatment with DEHP kept the serum LPS at the similar level as the control group (*P* = 0.66). This indicated that *L. plantarum* TW1-1 would prevent the increase of intestinal permeability caused by DEHP exposure.

### Effects of *L. plantarum* TW1-1 on the Diversity and Abundance of Gut Microbiota in DEHP-Exposed Mice

**Figure 5:**
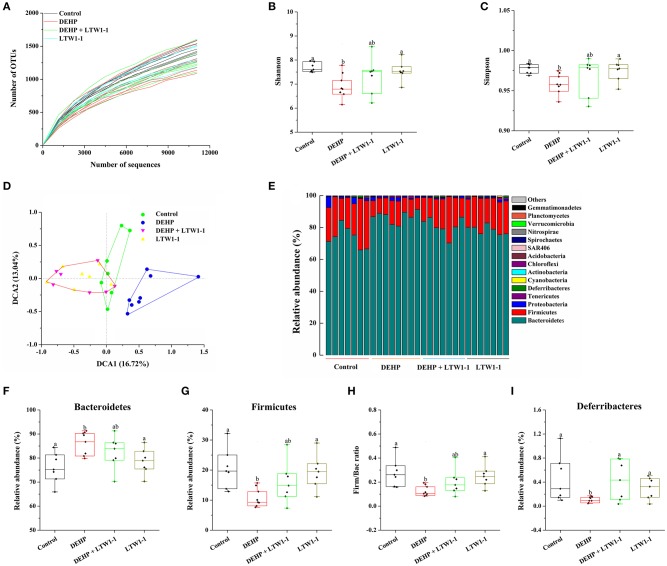
Effects of *L. plantarum* TW1-1 treatment on the structural changes and microbial diversity of the gut microbiota in DEHP-exposed mice. **(A)** Rarefaction curve. **(B)** Bacterial diversity as assessed by the Shannon index. **(C)** Bacterial diversity as assessed by the Simpson index. **(D)** DCA of fecal microbiota in the four groups. **(E)** Relative abundance of each fecal microbial profile at the phylum level in the four groups. **(F,G,I)** Relative abundances of significantly changed bacterial phyla (*Bacteroidetes, Firmicutes*, and *Deferribacteres*). **(H)** The Firm/Bac ratio in the different groups. The data are expressed as mean ± S.E.M. (*n* = 7–8), different letters represent significant differences between groups by Tukey's test (*P* < 0.05).

We performed Miseq sequencing analysis of 16S rRNA to determine the diversity and abundance of gut microbiota associated with DEHP and/or *L. plantarum* TW1-1 treated mice at baseline and at the end of the experiment. Although environmental factors, such as house conditions and dietary, change the overall composition of the gut microbiota in humans and animals observed in previous study (Nguyen et al., 2015). In the present study, at baseline, our results showed that there was no significant difference observed in the diversity and abundance of gut microbiota between the four groups (Figure S1), indicating that environmental factors do not induce the difference in the mouse gut microbiota between four groups before intervention. However, the diversity and abundance of gut microbiota were difference between DEHP group and other three groups at the end of the experiment (Figure 5). The alpha-diversity assessed by rarefaction curve, Shannon, or Simpson index, showed that bacterial species diversity of the DEHP-exposed mice was lower than that of control group (*P* < 0.01), which was restored by *L. plantarum* TW1-1 treatment (Figures 5A–C). DCA data showed that phylogenetic community structures were significantly different between DEHP-exposed samples and others, and the DEHP group was clearly separated from other groups along DCA1 (16.72%) (Figure 5D), suggesting that DEHP exposure significantly changed gut microbiota composition and *L. plantarum* TW1-1 supplement diminished the effect of DEHP on microbiota changes. Figure 5E showed the bacterial composition and the relative abundance at the phylum level of each individual sample in four groups. The dominant phyla in the gut microbiota were *Bacteroidetes, Firmicutes, Proteobacteria, Tenericutes*, and *Deferribacteres* (relative abundance > 0.5%). Comparing the DEHP group to the control mice, there was a significant increase in relative abundance of *Bacteroidetes* (Figure 5F; 86.4 vs. 76.0%, *P* < 0.01) and reduction in *Firmicutes* and *Deferribacteres* (Figures 5G,I; *P* < 0.01 and *P* < 0.05, respectively). *L. plantarum* TW1-1 treatment significantly attenuated DEHP-induced increase in *Bacteroidetes* and decreases in *Firmicutes* and *Deferribacteres*. The ratio of *Firmicutes* to *Bacteroidetes* (Firm/Bac ratio) was significantly decreased upon DEHP exposure (*P* = 0.004), which was mitigated by *L. plantarum* TW1-1 treatment (Figure 5H). The major composition and relative abundance of gut microbiota at the class, order, family, and genus levels in mice feces were also presented (relative abundance > 0.5%) (Figure S2). At the genus level (Figure S2D), the results showed that the relative abundance of *Prevotella* increased after DEHP exposure (*P* < 0.001), while that of *Oscillospira, Paraprevotella, Coprococcus, Lactobacillus, Ruminococcus*, and *Mucispirillum* decreased (*P* < 0.05). *L. plantarum* TW1-1 administration countered DEHP-induced *Prevotella* abundance increase and *Oscillospira, Paraprevotella, Coprococcus, Lactobacillus, Ruminococcus*, and *Mucispirillum* decreases.

**Figure 6:**
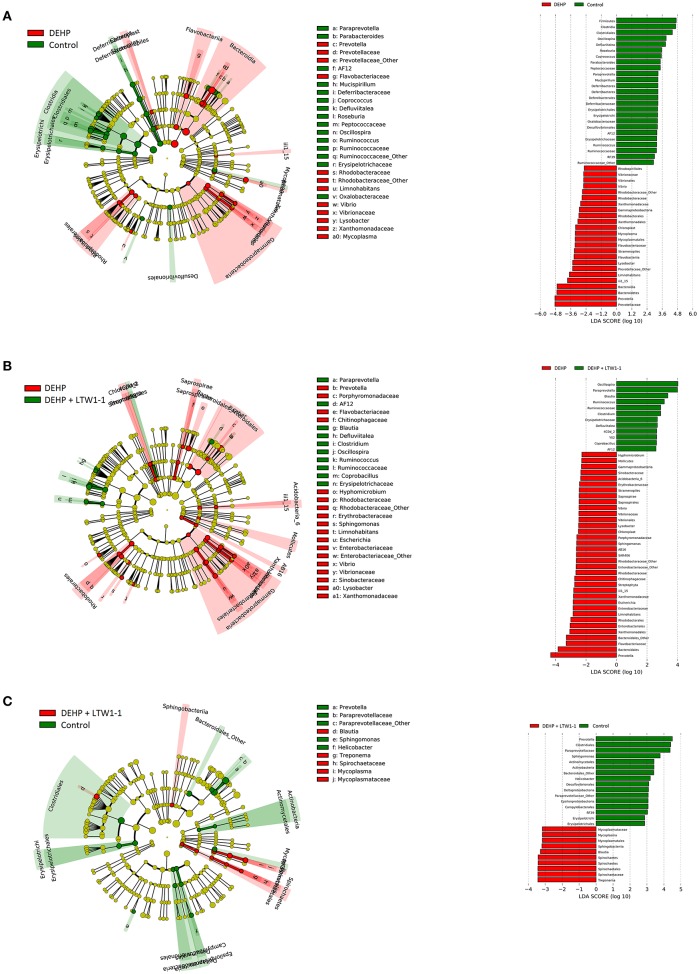
Cladogram and corresponding LDA scores showed the most differentially abundant taxa in gut microbiota from phylum to genus. **(A)** The taxa most highly associated with DEHP (red) or Control (green) and **(B)** DEHP (red) or DEHP + LTW1-1(green) and **(C)** DEHP + LTW1-1(red) or Control (green). Circle sizes in the cladogram plot are proportional to bacterial abundance. The circles represent, going from the inner circle to the outer circle: phyla, genus, class, order, and family.

Furthermore, cladograms generated from LEfSe analyses and corresponding LDA scores showed the most differentially abundant taxa in gut microbiota from phylum to genus and provided phylogenic information (Figure 6). The *prevotella* genus of the *Bacteroidetes* phylum was over-represented in DEHP-exposed mice as well as the *Rhodobacteraceae* family and the *Vibrio* and *Lysobacte*r genera of the *Proteobacteria* phylum (Figure 6A), whereas the *Mucispirillum* genus of the *Deferribacteres* phylum and the *Ruminococcaceae* and *Erysipelotrichaceae* families of the *Firmicutes* phylum were under-represented compared to those of the control group mice. Moreover, Figure 6B showed that *L. plantarum* TW1-1 treatment prevented the changes in taxa composition induced by DEHP exposure, although the *Escherichia* genus of the *Proteobacteria* phylum was still over-represented. Additionally, Compared with the control mice, the results showed only very few differences in gut microbiota between DEHP + LTW1-1 group and the Control group mice (Figure 6C), suggesting that *L. plantarum* TW1-1 restored the gut mcirobita of DEHP-exposed mice to that of the control mice. These data indicate that these bacteria, including *prevotella* genus, *Rhodobacteraceae* family, *Vibrio* family, *Lysobacte*r families, *Mucispirillum* genus, *Ruminococcaceae* family, and *Erysipelotrichaceae* family, were sensitive to DEHP and involved in the protective effects of *L. plantarum* TW1-1 against DEHP exposure. In summary, these results suggest that DEHP destroyed the balance of gut microbiota composition and *L. plantarum* TW1-1 had the ability to improve it.

### Correlation Between Gut Microbiota and Testicular Damage Parameters

**Figure 7:**
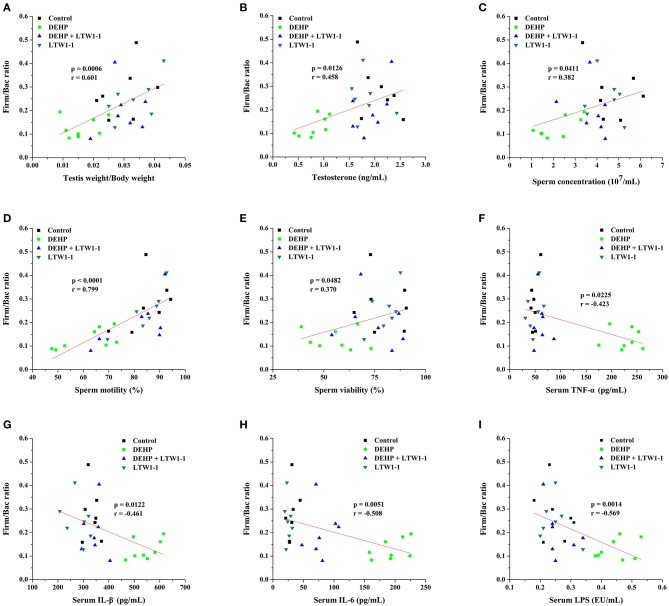
Spearman correlation analysis between the Firm/Bac ratio and testicular function parameters and inflammation were performed. The data were determined by Spearman's rho, which correspond to the “*r*” and “*p*” values, respectively, as shown in each plot. The Y-axis indicates the Firm/Bac ratio for each mouse compared with Relative testis weight **(A)**, Testosterone **(B)**, Sperm concentration **(C)**, Sperm motility **(D)**, Sperm viability **(E)**, TNF-α **(F)**, IL-1β **(G)**, IL-6 **(H)**, and LPS **(I)** (*n* = 7–8).

Spearman's correlation analysis was used to determine associations between key gut microbial taxa and testicular function (including relative testis weight, testosterone, and sperm activity) (Table 2). The *prevotella* and *Bacteroidales* in the *Bacteroidetes* phylum were negatively associated with relative testis weight, testosterone, sperm concentration and viability, respectively. S24–7 and *Paraprevotella* in the *Bacteroidetes* phylum and *Mucispirillum* in the *Deferribacteres* phylum were positively correlated with testosterone and sperm activity. Bacteria from *Firmicutes* that were positively correlated with testicular function were restored by *L. plantarum* TW1-1 pretreatment in DEHP-exposed mice, including *Oscillospira, Lachnospiraceae, Coprococcus, Lactobacillus, Ruminococcus*. These results indicate that *Bacteroidetes, Deferribacteres*, and *Firmicutes* were significantly associated with DEHP-induced testicular damage. In addition, based on the crucial role of Firm/Bac ratio in maintaining host health, we also analyzed the relationship between Firm/Bac ratio and testicular function and inflammation. Our data showed that Firm/Bac ratio was positively correlated with testicular health and negatively correlated with inflammation (including serum TNF-α, IL-β, IL-6, and serum LPS) (Figure 7). All together, these findings indicate that gut microbiota was significantly correlated with DEHP-induced testicular damage and systemic inflammatory response.

**Table 2:** Spearman's correlation between key gut microbial taxa and testicular damage parameters in DEHP and/or *L. plantarum* TW1-1treated mice.

	**Testis weight/body weight**		**Testosterone**		**Sperm concentration**		**Sperm motility**		**Sperm viability**	
	***r***	***p***	***r***	***p***	***r***	***p***	***r***	***p***	***r***	***p***
*Prevotella*	−0.343	0.0687	**−0.505**	**0.0052**	−0.354	0.0600	−0.329	0.0815	−0.266	0.1626
S24-7	0.263	0.1679	0.103	0.5950	**0.437**	**0.0178**	0.208	0.2786	0.307	0.1053
*Bacteroides*	−0.055	0.7769	0.133	0.4910	−0.019	0.9222	0.020	0.9172	0.051	0.7918
*Clostridiales*	0.234	0.2228	0.230	0.2303	0.086	0.6584	0.093	0.6364	0.058	0.7635
*[Prevotella]*	−0.306	0.1067	−0.049	0.7996	−0.120	0.5353	−0.287	0.1319	0.055	0.7781
*Oscillospira*	**0.585**	**0.0009**	**0.514**	**0.0043**	0.287	0.1315	0.334	0.0770	0.297	0.1175
*Lachnospiraceae*	**0.460**	**0.0121**	0.365	0.0514	0.278	0.1442	0.273	0.1523	0.313	0.0984
*Rikenellaceae*	−0.150	0.4384	0.161	0.4052	−0.024	0.9001	−0.026	0.8920	−0.054	0.7801
*Paraprevotella*	0.342	0.0696	**0.411**	**0.0269**	**0.451**	**0.0140**	**0.499**	**0.0058**	**0.397**	**0.0331**
*Bacteroidales*	**−0.466**	**0.0108**	−0.170	0.3779	**−0.452**	**0.0138**	−0.221	0.2488	**−0.474**	**0.0094**
*Parabacteroides*	−0.137	0.4795	0.325	0.0857	0.188	0.3292	0.098	0.6126	0.203	0.2899
*Bacteroidales*	−0.322	0.0882	−0.233	0.2234	−0.181	0.3466	−0.342	0.0695	−0.037	0.8480
*Lactobacillus*	0.024	0.9034	0.329	0.0814	**0.406**	**0.0290**	0.222	0.2472	0.314	0.0977
*Coprococcus*	0.325	0.0859	0.303	0.1101	0.276	0.1471	0.045	0.8167	**0.387**	**0.0380**
*Mucispirillum*	**0.416**	**0.0249**	**0.505**	**0.0052**	0.279	0.142	**0.429**	**0.0203**	0.161	0.4048
*Ruminococcus*	**0.635**	**0.0002**	0.361	0.0546	**0.568**	**0.0013**	**0.563**	**0.0015**	**0.48**	**0.0084**

*Spearman's “r” and “P <0.05” highlighted in bold are statistically significant*.

## Discussion

Probiotics have become a new therapeutic approach for series of diseases. Our previous study has reported that *L. plantarum* TW1-1 has anti-inflammatory and anti-oxidative stress activities. In the current study, we examined the effect of *L. plantarum* TW1-1 treatment on DEHP-induced testicular injury in adult male mice. Our research confirmed for the first time that *L. plantarum* TW1-1 prevented DEHP-induced testicular injury in mice via modulating gut microbiota and decreasing inflammation.

Previous studies have demonstrated that DEHP induces testicular damage and adversely affects reproductive health in male rats (Zhang et al., 2014; Abdel-Kawi et al., 2016). In the present study, DEHP exposure induced testicular toxicity which was represented by the decrease in the weight of testes, sperm activity and testosterone secretion, coinciding with previous studies (Abdel-Kawi et al., 2016). Similarly, other studies have also verified that DEHP and its metabolites inhibits testosterone levels and decreases the relative weight of testes by disturbing hypothalamic-pituitary-testis axis and ERK-mediated 5α-Reductase 2 (Botelho et al., 2009; Ha et al., 2016). Our results demonstrated that *L. plantarum* TW1-1 treatment significantly increased the weight of testes as well as sperm activity and the serum testosterone levels reduced by DEHP exposure, suggesting that *L. plantarum* TW1-1 protected against DEHP-induced testicular injury. Furthermore, histopathological analyses showed that DEHP exposure reduced the thickness of the germinal epithelium and induced irregular arrangement of germ cells in the seminiferous tubules with exfoliation of these cells into the lumen, which was consistent with the findings of previous studies (Zhang et al., 2014; Abdel-Kawi et al., 2016). Importantly, we observed there were no changes of germ cells and seminiferous tubules between control group and DEHP + LTW1-1 group, indicating that *L. plantarum* TW1-1 alleviated DEHP-induced testicular toxicity.

In response to DEHP toxicity, some ROS are generated disturbing the activity of antioxidant enzyme (Wang et al., 2012; Abdel-Kawi et al., 2016). High levels of ROS caused LPO of outer membrane in sperm causing the loss of motility simultaneously affecting sperm concentration (Urata et al., 2001). GSH, CAT and SOD are considered to be important factors in the protection of host organisms. The accumulation of LPO products decreases antioxidant activity, and increases free radical production resulting in an oxidative stress. MDA, as an end product of LPO, is one of the most popular and reliable oxidative stress markers, the accumulation of which decreases antioxidant activity, and increases free radical production resulting in oxidative stress. It has been reported that DEHP and its metabolites mono-(2-ethylhexyl) phthalate (MEHP) and 2-ethylhexanol (2 EH) induce oxidative stress in the testes, leading to disrupting the function of the Sertoli cell and Leydig cell associating with the apoptosis of spermatocytes (Kasahara et al., 2002; Richburg et al., 2002; Somasundaram et al., 2017). Therefore, oxidative stress is recognized as a critical mechanism underlying DEHP-induced testicular damage. In accordance with previous studies (Abd El-Fattah et al., 2016), our results demonstrated that DEHP reduced GSH concentration, SOD and CAT activities, while increased LPO and MDA concentrations in the serum and testes of mice, indicating that DEHP accelerated the oxidative stress. This suggests that DEHP-induced testicular damage is correlated with the reduction of antioxidant activity and increase of lipid peroxidant. Furthermore, we also found that *L. plantarum* TW1-1 restored the concentrations of GSH, LPO, and MDA, the activities of SOD and CAT in the serum and testes of the DEHP-exposed mice to those of the control mice. These results indicate pretreatment of *L. plantarum* TW1-1 alleviates DEHP-induced reproductive toxicity via regulating oxidative stress.

To further evaluate the protective effects of *L. plantarum* TW1-1 on DEHP-induced testicular injury, inflammatory responses in the testes were also examined. Oxidative stress has been demonstrated to up-regulate inflammatory responses in the testes though increasing the secretion of inflammatory factors such as TNF-α and IL-6, indicating that inflammatory responses is associated with apoptosis of spermatogenic cell and aggravates testicular injury (Sherif et al., 2014; Hussein et al., 2015). In human macrophages, DEHP treatment increases the production of inflammatory cytokines, such as TNF-α, IL-1β, IL-8, and IL-6, which induces the inflammatory response during allergic reactions (Nishioka et al., 2012). DEHP promotes the development of immune responses and subsequent male infertility through increasing the expression of inflammatory mediators (Larsen et al., 2007; Hirai et al., 2015). Recent studies have reported that DEHP increased the production of inflammatory cytokines such as TNF-α and IL-1β in mice testes (Bahrami et al., 2018a,b). In agreement with previous studies, our results showed that DEHP elevated serum TNF-α, IL-1β, and IL-6 levels and increased the mRNA expression of TNF-α, IL-1β, and IL-6 in the testes. This indicates that DEHP exposure increases the expression of inflammatory mediators in mice. In addition, we found that *L. plantarum* TW1-1 significantly decreased serum TNF-α, IL-1β, and IL-6 levels and the gene expression of these inflammatory factors in the testes, suggesting that *L. plantarum* TW1-1 could attenuate systemic and testicular inflammatory responses. Our previous study have revealed the anti-inflammatory effects of *L. plantarum* TW1-1 (Wu et al., 2017). Therefore, these findings indicate that *L. plantarum* TW1-1 prevents DEHP-induced testicular damage via inhibiting inflammatory response.

Furthermore, we determined the effect of *L. plantarum* TW1-1 on DEHP-induced intestinal dysfunction. Our results confirmed for the first time that DEHP exposure reduced the concentrations of GSH, LPO, and MDA, the activities of SOD and CAT, and increased gene expression of TNF-α, IL-1β, and IL-6 in the colon. This suggests that DEHP induce oxidative stress and inflammatory response in the colon. Over production of ROS has been demonstrated to cause chronic intestinal inflammatory diseases by increasing gut permeability and infiltration of inflammatory leukocytes into intestine. Intestinal oxidative stress is recognized as a major contributor to intestinal injury, resulting in endotoxin translocation (Assimakopoulos et al., 2004). Previous study reported that DEHP exposure induces colon tumor promotion in rats (Chen et al., 2017). The pulmonary inflammation in rat offspring were exacerbated when pregnant or lactating rats were exposed to DEHP with the increased inflammatory factor IL-4 (Wang et al., 2018). Thus, our data suggest that DEHP-induced colonic injury could be related with oxidative stress and inflammatory response in the colon. In addition, we also found DEHP increased the level of the serum gut-derived bacterial product LPS and intestinal permeability. LPS and intestinal hyper-permeability are involved in various diseases. Therefore, these results verify that DEHP exposure induces colonic injury and results in intestinal dysfunction via inducing oxidative stress and inflammatory response in the colon. It has been demonstrated that some probiotics can reduce oxidative stress and liver injury by increasing GSH level (Lutgendorff et al., 2007; Wu et al., 2017). The probiotic strain, *Lactobacillus Fermentum*, releases colonic GSH preventing the inflammatory processes in rat (Peran et al., 2006). Inflammation can be reduced *in vitro* or colitis mice by probiotic strains, like *Lactobacillus* and *Bifidobacterium* (Roselli et al., 2006; Veiga et al., 2010). Previous study demonstrated that probiotics significantly prevents pro-inflammatory (CD11c^+^, MMP-12^+^) macrophages permeating into adipose tissue and attenuates TNF-α expression (Wang et al., 2015b). Our previous study revealed that *L. plantarum* TW1-1 treatment alleviates inflammatory responses as a result of induction by chromium exposure in mice (Wu et al., 2017). In the present study, *L. plantarum* TW1-1 pretreatment recovered the concentrations of GSH, LPO, and MDA, the activities of SOD and CAT, and restrained the expressions of TNF-α, IL-1β, and IL-6 in the colon of DEHP-exposed mice. This indicates that *L. plantarum* TW1-1 can inhibit inflammation and oxidative response induced by DEHP exposure in the colon. As previously observed, probiotic strains improves mucosal barrier homeostasis and restores gut barrier permeability (Martín et al., 2016). *Bifidobacterium lactis* CNCM I-2494 restrains intestinal permeability in rats (Agostini et al., 2012). *Lactobacillus casei* improves the anti-oxidative and anti-inflammatory capacities to struggle with endotoxin-induced liver injury in rats (Wang et al., 2013). Similar to these previous studies, our results indicate that *L. plantarum* TW1-1 alleviate intestinal dysfunction induced by DEHP exposure.

Intestinal dysfunction, including gut leaky and gut dysbiosis, causes translocation of intestinal bacteria or their metabolites from the digestive tract to the systemic circulation, resulting in several diseases (Lichtman et al., 1990; Yan et al., 2011). Emerging studies have demonstrated that the gut microbiota might be related to gut inflammatory responses (Cani et al., 2009), which participate in a series of diseases and regulates host health. According to a literature, gut microbiota may affect the development of obesity and diabetes through controlling the changes of environmental factors (Snedeker and Hay, 2012). Although the exact mechanism for the change in gut microbiota composition is unknown, the destruction of gut microbial community maybe aroused by drugs, diet, or environmental pollutants (Jin et al., 2017). Recent evidence confirmed that environmental pollutants induces colonic inflammation, disturbs the host microbial ecosystem and ultimately affects human health (Jin et al., 2016). To investigate the relationship between DEHP-induced testicular injury and gut microbiota, fecal bacterial 16S rRNA sequencing was performed. MiSeq results showed that DEHP exposure significantly reduced the diversity and abundance of gut microbiota compared to those of the control group mice, which was recovered by *L. plantarum* TW1-1 treatment. As revealed by DCA, *L. plantarum* TW1-1 resulted in the gut microbiota of DEHP-exposed mice very similar to that of the control mice, suggesting that *L. plantarum* TW1-1 effectively improve the gut microbiota disturbed by DEHP exposure. Moreover, at the phylum level, DEHP significantly increased the relative abundance of *Bacteroidetes*, whereas decreased that of *Firmicutes* and *Deferribacteres*. However, *L. plantarum* TW1-1 pretreatment significantly attenuated DEHP-induced increase in *Bacteroidetes* and decreases in *Firmicutes* and *Deferribacteres*. As predominant members, *Firmicutes* and *Bacteroidetes*, impart key functions to their host, such as metabolism and developmental and immunologic properties (Mazmanian et al., 2005). Previous study has demonstrated that the ratio of Firm/Bac is positively related to obesity in mammals (Turnbaugh et al., 2009). In the present study, DEHP markedly decreased the ratio of Firm/Bac, consistent with previous findings that DEP increased the *Bacteroidetes* populations and significantly decreased *Firmicutes* populations (Hu et al., 2016). Similarly, tetrachlorodibenzofuran treatment significantly altered *Bacteroidetes* populations compared with control group, and reduced the ratio of Firm/Bac (Zhang et al., 2015a). However, other studies demonstrated that the gut microbiota of obese humans and high-fat diet-fed mice represented high Firm/Bac ratio (Ley et al., 2006; Brun et al., 2007). Generally, the Firm/Bac ratio is crucial to maintaining host health. In the current study, correlation analysis between Firm/Bac ratio and reproductive health parameters revealed that Firm/Bac ratio was positively correlated with relative testis weight and testosterone level as well as sperm activity. Therefore, our findings indicate that *L. plantarum* TW1-1 attenuates DEHP-induced testicular injury probably associated with the change of the Firm/Bac ratio.

At the genus level, DEHP exposure increased the relative abundance of *Prevotella* and *Paraprevotella*, and decreased that of *Oscillospira, Coprococcus, Lactobacillus, Ruminococcus*, and *Mucispirillum*. However, *L. plantarum* TW1-1 prevented DEHP-induced *Prevotella* and *Paraprevotella* increases and *Oscillospira, Coprococcus, Lactobacillus, Ruminococcus*, and *Mucispirillum* decreases. Furthermore, LEfSe analyses revealed that *prevotella* genus, *Rhodobacteraceae* family, *Vibrio* family, *Lysobacte*r families, *Mucispirillum* genus, *Ruminococcaceae* family and *Erysipelotrichaceae* family, were sensitive to DEHP and involved in the protective effects of *L. plantarum* TW1-1 against DEHP exposure. Similarly, DEP treatment results in an increase in abundance of *Prevotella* genus and a decrease in that of *Lactobacillus* genus (Hu et al., 2016). In addition, correlation analysis between gut microbiota and testicular damage identified that *prevotella* and *Bacteroidales* were negatively associated with testicular function, while S24-7, *Paraprevotella, Mucispirillum, Oscillospira, Lachnospiraceae, Coprococcus, Lactobacillus*, and *Ruminococcus* were positively correlated with that. *L. plantarum* TW1-1 pretreatment reduced the increases of *prevotella* and *Bacteroidales*, and elevated the decreases of S24–7, *Paraprevotella, Mucispirillum, Oscillospira, Lachnospiraceae, Coprococcus, Lactobacillus*, and *Ruminococcus* in DEHP-exposed mice. This suggests that gut microbiota is significantly correlated with DEHP-induced testicular damage. It has been reported that *Prevotella, Clostridiales, Coprococcus, Lactobacillus*, and *Ruminococcus* are associated with human health and disease. *Prevotella* is reported to promote inflammatory disease by inducing neutrophil dysfunction (Matsui et al., 2014). *Lactobacillus* motivates the host to produce immunoglobulins and improve host immunity against gastrointestinal infections (Sanz et al., 2007). The abundance of pathogenic bacteria increases, while that of beneficial bacteria decreases, which may contribute to intestinal dysfunction and result in diseases. Overall, the present study demonstrates that *L. plantarum* TW1-1 treatment maintains gut bacterial balance by modulating gut microbiota composition in DEHP-exposed mice.

Because of gut dysbiosis and hyper-permeability, gut-derived bacteria and bacterial endotoxin, are easily delivered to the systemic circulation, leading to varies of diseases. LPS (endotoxin), a major component of the gram-negative bacterial cell wall, results in activation of inflammatory cells and production of proinflammatory cytokines, such as TNF-α, IL-1β. In the current study, the serum LPS level was positively correlated with intestinal permeability (Figure S3), which indicated that the translocation of LPS into the blood circulatory system was associated with increased intestinal permeability. Moreover, DEHP exposure destroyed the balance of gut microbiota and reduced the ratio of Firm/Bac in DEHP-exposed mice, which was alleviated by pretreatment of *L. plantarum* TW1-1. Correlation analysis results showed that the ratio of Firm/Bac was negatively correlated with serum inflammatory factors and LPS, suggesting that alterations in gut microbial composition and gut dysbiosis were related to increased intestinal permeability and inflammatory responses. Taken together, out data indicate that *L. plantarum* TW1-1 alleviates DEHP-induced testicular injury maybe associated with gut dysbiosis.

## Conclusion

**Figure 8:**
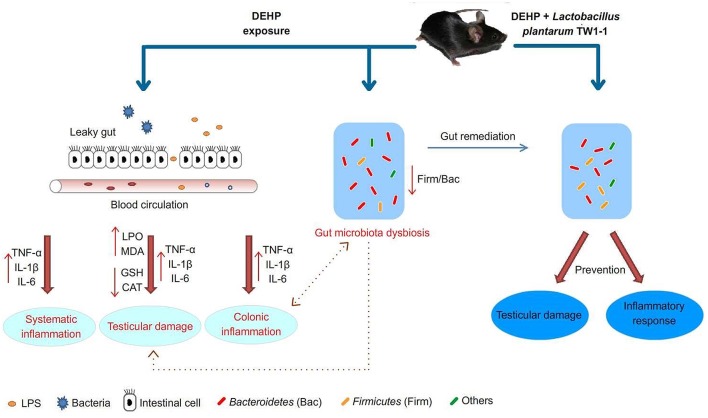
Proposed pathways of protection by *L. plantarum* TW1-1 treatment against DEHP-induced testicular damage.

In summary, our results showed that DEHP exposure induced gut microbiota dysbiosis, which may be correlated with DEHP-induced testicular toxicity. The effects and possible underlying mechanisms of *L. plantarum* TW1-1 blocking DEHP-induced testicular damage are briefly depicted in Figure 8. Although *L. plantarum* TW1-1 did not reduce the residual DEHP concentration in mice, we provide the initial evidence that *L. plantarum* TW1-1 modulated gut microbiota and effectively ameliorated DEHP-induced testicular injury via anti-oxidative and anti-inflammatory capacities. Based on the relationship between environmental pollutants and gut microbiota, gut remediation of microbial communities associated with reproductive disease could be useful for prevention or therapy.

## Ethics Statement

This study was carried out in accordance with the recommendations of the Guide for the Care and Use of Laboratory Animals and the Declaration of Helsinki. The protocol was approved by the United States National Institutes of Health.

## Author Contributions

XT and XL designed this study. PF, ZeY, and RL performed the experiments. Data were analyzed by ZhY in collaboration with XT and PF. XT wrote the manuscript. JL, JH, ZhY, AK, PL, and XL contributed to writing by providing suggestions and helping in revision. All authors reviewed and approved the final version of the manuscript.

### Conflict of Interest Statement

The authors declare that the research was conducted in the absence of any commercial or financial relationships that could be construed as a potential conflict of interest.

## Abbreviations

*L. plantarum* TW1-1: *Lactobacillus plantarum* TW1-1
DEHP: Diethylhexylphthalate
TNF-α: Tumor necrosis factor-α
IL: Interleukin
LPS: Lipopolysaccharide
Firm/Bac: Firmicutes to Bacteroidetes
DEP: Diethyl phthalate
GSH: Glutathione
LPO: Lipid peroxidation
MDA: Malondialdehyde
SOD: Super oxide dismutase
CAT: Catalase
ELISA: Enzyme-linked immunosorbent assay
OTUs: Operational taxonomic units
DCA: Detrended correspondence analysis
ROS: Reactive oxygen species.
