# Supplementary material for: Lactobacillus plantarum TW1-1 Alleviates Diethylhexylphthalate-Induced Testicular Damage in Mice by Modulating Gut Microbiota and Decreasing Inflammation
Source: Front Cell Infect Microbiol. 2019 Jun 26;9:221. doi: 10.3389/fcimb.2019.00221 (PMC6607032; doi:10.3389/fcimb.2019.00221)
Supplement: Supplementary file 1 [file Data_Sheet_1.PDF]

Supplementary Material

Lactobacillus plantarum TW1-1 alleviates diethylhexylphthalate-induced testicular damage in mice by modulating gut microbiota and decreasing inflammation

Xiao-zhu Tian[a,1], Zheng-sheng Yu[a,1], Peng-ya Feng[a], Ze Ye[a], Rong Li, Ju-yuan Liu[a], Jun-ping Hu[b], Pu Liu[a], Xiang-kai Li[a,*]

a Ministry of Education Key Laboratory of Cell Activities and Stress Adaptations, School of Life Science, Lanzhou University, 222 Tian Shui South Road, Lanzhou, Gansu 730000, P.R.China
b Key Laboratory for Reproductive Medicine and Embryo, the Reproductive Medicine Special Hospital of the First Hospital of Lanzhou University, Lanzhou, Gansu 730000, P.R.China.

1 The authors who contributed equally to this study

*Correspondence:
Dr. Xiangkai Li
xkli@lzu.edu.cn

Table S1

Effects of *L. plantarum* TW1-1 treatment on the body weight and testis weight of DEHP-induced mice at the end of experiment.

	Control	DEHP	DEHP + LTW1-1	LTW1-1
Testis weight (g)	1.053 ± 0.121[a]	0.613 ± 0.094[b]	0.934 ± 0.085[a]	1.187 ± 0.108[a]
Body weight (g)	35.26 ± 3.61[a]	37.55 ± 3.22[a]	35.92 ± 4.76[a]	37.09 ± 4.28[a]
tw/bw	0.029 ± 0.002[a]	0.016 ± 0.002[b]	0.027 ± 0.002[a]	0.032 ± 0.003[b]

The data are expressed as mean ± S.E.M. (n = 8), different letters represent significant differences between groups by Tukey's test (P < 0.05). Body weight: bw, Testis weight: tw.

Table S2

The primer sequence used in this study.

DNA sample	Forward primer (5' to 3')	Reverse primer (5' to 3')
Fecal DNA (total bacteria)	GTGCCAGCMGCCGCGGTAA	CCCCGYCAATTCMTTTRAGT
GAPDH (mouse gene)	GTGTTCCTACCCCCAATGTGT	ATTGTCATACCAGGAAATGAGCTT
TNF-α	GTCTACTGAACTTCGGGGTGAT	GGCTACAGGCTTGTCACTCG
IL-1β	CCAACAAGTGATATTCTCCATGAG	ACTCTGCAGACTCAAACTCCA
IL-6	CTCTGCAAGAGACTTCCATCC	GAATTGCCATTGCACAACTC

TNF, Tumor Necrosis Factor; IL, Interleukin.

Table S3

Changes in serum GSH, CAT, SOD, LPO, and MDA levels in the different groups.

Groups	Serum index				
	GSH (mg/mL)	CAT (U/mL)	SOD (U/mL)	LPO (mmol/mL)	MDA (nmol/mL)
Control	6.37 ± 0.74[a]	26.51 ± 3.47[a]	27.63 ± 3.14[a]	1.05 ± 0.13[a]	2.99 ± 0.42[a]
DEHP	3.25 ± 0.46[b]	13.17 ± 2.16[b]	15.66 ± 1.14[b]	1.87 ± 0.11[b]	6.47 ± 0.32[b]
DEHP + LTW1-1	5.78 ± 0.48[a]	31.25 ± 4.54[a]	25.19 ± 2.02[a]	1.31 ± 0.15[a]	3.21 ± 0.45[a]
LTW1-1	6.21 ± 0.35[a]	28.48 ± 3.18[a]	30.17 ± 3.26[a]	1.08 ± 0.14[a]	2.77 ± 0.34[a]

The data are expressed as mean ± S.E.M. (n = 8), different letters represent significant differences between groups by Tukey's test (P < 0.05).

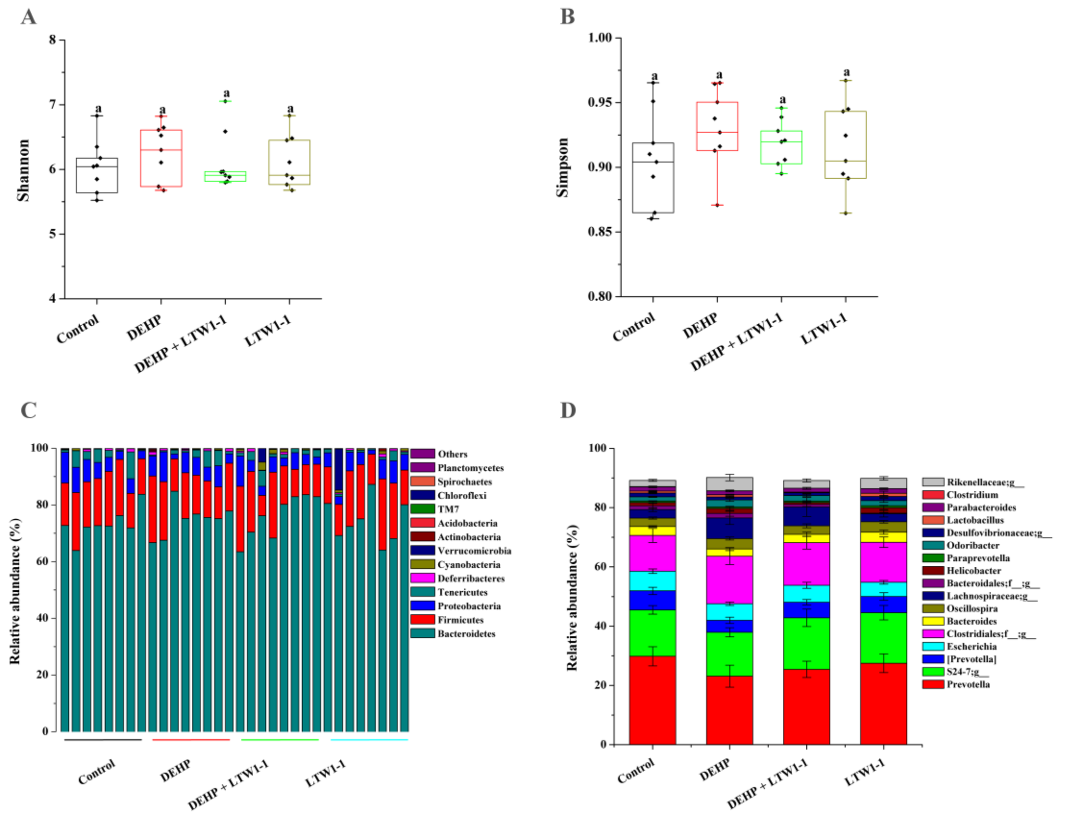

Fig. S1 Bacterial 16S rRNA-based analysis of the gut microbiota in the four group mice at baseline. (A) Bacterial diversity as assessed by the Shannon index. (B) Bacterial diversity as assessed by the Simpson index. (C) Relative abundance of each fecal microbial profile at the phylum level. (D) Relative abundance of fecal microbiota at the genus level. The data are expressed as mean ± S.E.M. (n = 8), different letters represent significant differences between groups by Tukey's test (*P* < 0.05).

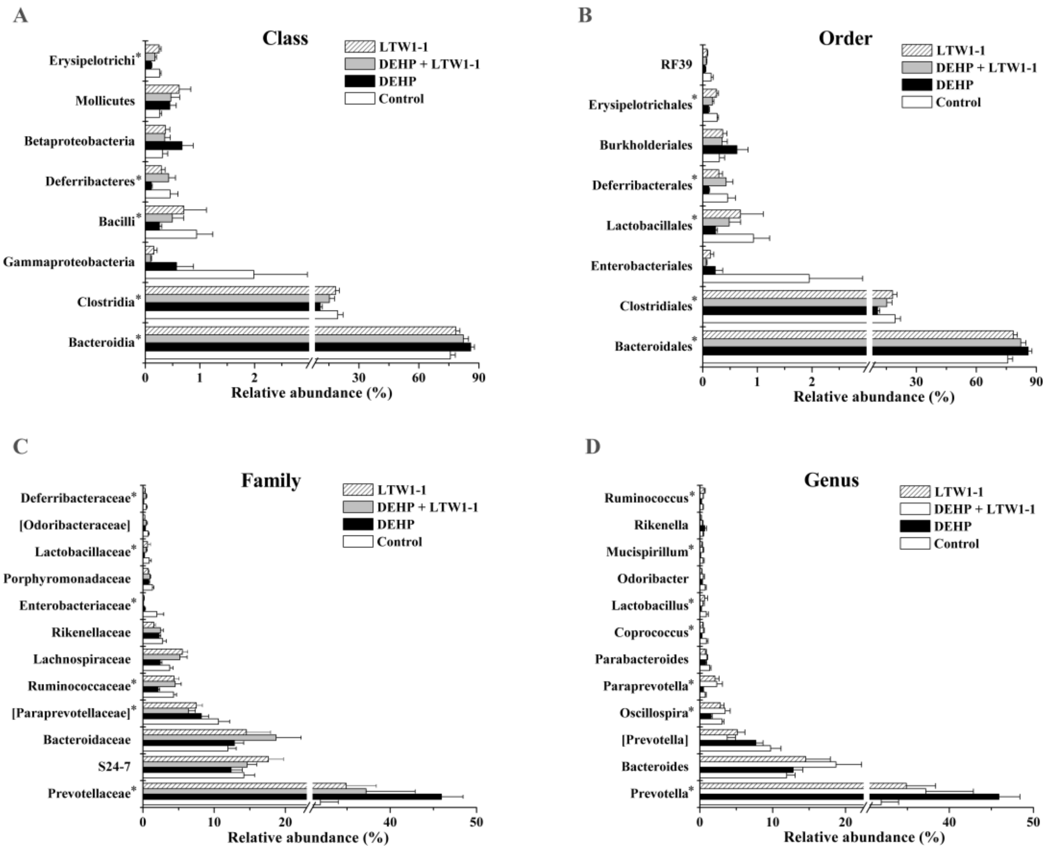

Fig. S2 The major composition of gut microbiota in mice feces during DEHP and/or *L. plantarum* TW1-1 treatment. (A) Relative abundance at the class level. (B) Relative abundance at the order level. (C) Relative abundance at the family level. (D) Relative abundance at the genus level. (D) Relative abundance at the genus level. The data are expressed as mean ± S.E.M. (n = 7–8), **P* < 0.05: DEHP group vs. other groups by ANOVA followed by Tukey's test.

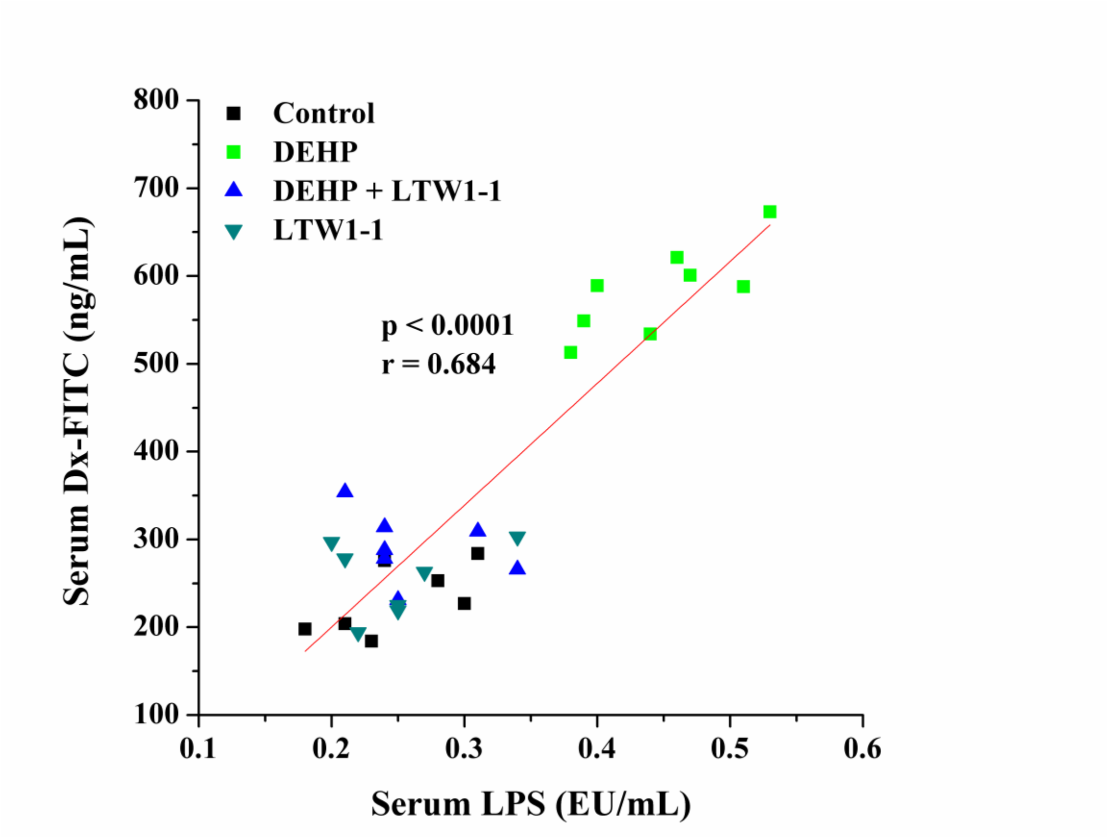

Fig. S3 Spearman correlation analysis between serum Dx-FITC levels and serum LPS levels. The data were determined by Spearman's rho, which correspond to the "r" and "p" values, respectively, as shown in each plot. The Y-axis indicates serum Dx-FITC levels for each mouse compared with serum LPS levels, (n = 7–8).
